# Co-BDC MOF/Graphene Nanohybrid as an Efficient Electrode Material for High-Performance Supercapacitors

**DOI:** 10.1021/acsomega.5c11771

**Published:** 2026-03-13

**Authors:** Ana L. Braga, Tomaz A. S. Lima, Victor D. S. Fortunato, Danielle D. Justino, Pedro G. R. Gomes, Rayane C. F. Silva, Larissa F. M. A. Vieira, Hélio Ribeiro, Ana Paula C. Teixeira, Paulo F. R. Ortega, Rodrigo L. Lavall, Raquel V. Mambrini, João Paulo C. Trigueiro

**Affiliations:** † Departamento de Química, Centro Federal de Educação Tecnológica de Minas Gerais, 74355 CEFET-MG, Belo Horizonte, MG 30480-000, Brasil; ‡ Departamento de Química, Instituto de Ciências Exatas, 28114 Universidade Federal de Minas Gerais, UFMG, Belo Horizonte, MG 31270-901, Brasil; § Centro de Tecnologia em Nanomateriais e Grafeno (CTNano/UFMG), Universidade Federal de Minas Gerais, UFMG, Belo Horizonte, MG 31310-270, Brasil; ∥ Departamento de Engenharia de Materiais, Escola de Engenharia, Universidade Presbiteriana Mackenzie, São Paulo, SP 01307-012, Brasil; ⊥ Departamento de Química, Centro de Ciências Exatas, Universidade Federal de Viçosa, UFV, Viçosa, MG 36570-900, Brasil

## Abstract

A Co-BDC metal–organic framework/reduced graphene oxide (rGO) hybrid was synthesized via in-situ MOF growth followed by controlled pyrolysis to obtain a Co-BDC–derived nanoporous carbon (NPC)/rGO electrode for supercapacitors. The rGO-assisted transformation preserves the precursor integrity and yields a uniform, conductive, and porous carbon architecture with well-dispersed Co^0^ species. The optimized NPC/rGO-10 electrode delivers a specific capacitance of 172 F g^–1^ at 1 A g^–1^ with 96% retention after 6,000 cycles. An asymmetric device (NPC/rGO-10//rGO) shows stable operation over 30,000 cycles and maintains 94.4%, emphasizing the outstanding reversibility and structural robustness of the electrode material. Additionally, rGO incorporation reduced the level of Co leaching into the electrolyte, indicating enhanced chemical stability under electrochemical operation. The novelty of this work lies in the rGO-assisted MOF-to-carbon conversion strategy, which enables a robust nanoporous carbon/graphene hybrid with suppressed metal leaching and exceptional long-term stability.

## Graphical abstract

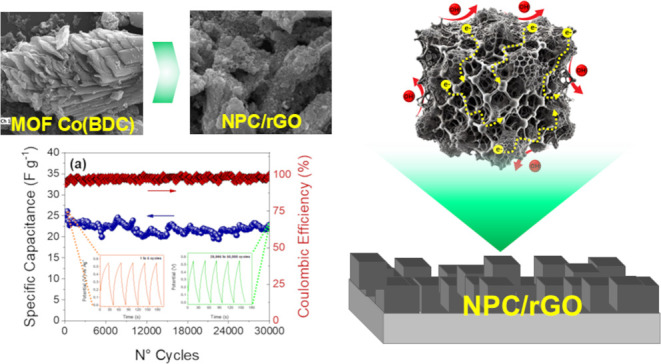

## 1 Introduction

The growing global demand for cleaner and more efficient energy sources has driven the development of energy-storage technologies that are not only effective but also sustainable. In this context, supercapacitors have emerged as promising devices, offering high power density, rapid charge and discharge cycles, and long service life, compared to conventional batteries.^1^ These characteristics make supercapacitors ideal for various applications, from portable electronics to electric vehicles, where consistent performance and reliability are critical. However, to expand their possibilities of use, it is necessary to overcome challenges related to their energy density, which is relatively low compared to batteries.^2^ Supercapacitors operate based on two main mechanisms: electric double-layer capacitance (EDLC), which depends on the separation of charges at the electrode–electrolyte interface, and pseudocapacitance, which involves rapid redox reactions on the surface of electrode materials, increasing storage capacity to considerable levels. When optimized, these two mechanisms can provide a unique combination of power and energy density in electrochemical storage.^3–5^ The performance of SCs is intrinsically linked to the electrode materials, which must combine a high surface area, excellent electrical conductivity, and the capability to perform redox reactions to maximize the efficiency of the charge and discharge processes. Among the most promising materials, carbon-based materials, such as graphene^6–8^ and carbon nanotubes, as well as transition metal oxides, such as MnO_2_, Fe_2_O_3_, and Co_3_O_4_, exhibit significant electrochemical potential due to their highly reactive structures.^9–11^

This field has made significant progress using metal–organic frameworks (MOFs). These porous crystalline and porous structures, composed of metal ions and organic ligands, provide remarkable flexibility in structural and functional customizations. MOFs have been extensively applied in several areas, including gas storage and delivery, catalysis, and chemical/biosensing, and more recently, they have demonstrated outstanding potential in energy-storage devices.^12–16^

Through controlled thermal treatment, MOFs can be converted into derivative materials that combine metal nanoparticles or metal oxides with conductive carbon matrices, providing significantly improved electrochemical properties.^17,18^ Such structural features promote efficient mass transport, enhanced exposure of active sites, and improved physicochemical stability, enabling the application of MOF-derived materials in catalysis,^19^ redox reactions,^20^ contaminant adsorption,^21^ and advanced environmental processes,^22^ where they outperform many conventional porous materials. Typically, the MOF thermal treatment is performed via two main approaches. The first is a two-step method that uses additional carbon sources, where MOFs serve as secondary carbon sources and sacrificial templates. Xu et al. were the first to introduce furfuryl alcohol (FA) into the micropores of MOF-5, employing a vapor-phase protocol as a sacrificial template for the synthesis of nanoporous carbons.^23^ The other method is the direct pyrolysis of MOFs, a simple one-step procedure that generally preserves the morphology of the original MOFs. Yamauchi’s group prepared nanoporous carbon through direct carbonization of Al-PCP, and the nanoporous carbon treated at 800 °C exhibited significantly higher porosity (surface area exceeding 5000 m^2^ g^–1^) than other carbon materials., This was the first comprehensive investigation of this approach.^24^

These approaches enable the development of hybrid materials that not only maximize capacitance and energy density but also improve stability and efficiency over long operating cycles. For example, the pyrolysis of structures such as ZIF-8 and ZIF-67 has generated hybrid materials with high performance in supercapacitors. These materials exhibited excellent specific capacitances, indicating their viability as electrode components for high-performance supercapacitors.^17,25–29^ Although significant progress has been made, several challenges remain to be addressed to enhance the capacitive performance. For example, carbon-based materials obtained by pyrolysis of MOFs primarily contain micropores, which limit the rate capability, particularly at elevated scan rates.^30^ As a result, electrolyte ions cannot fully saturate the inner pores of large-porosity materials within a limited time, thereby reducing their contribution to capacitance. Therefore, reducing the particle size of MOFs to facilitate ion transport, as well as combining them with highly conductive substances like graphene, is highly desirable to enhance the capacitive properties of nanoporous carbon derived from pyrolysis.^31–34^

The combination of graphene with nanoscale MOF-derived nanoporous carbons (NPCs) not only prevents the restacking of graphene by leveraging its surface area but also enables the creation of multidimensional conductive networks, which enhance charge transfer and improve the exposure of active sites, crucial factors for optimizing device performance. Based on the above considerations, we synthesized in situ a reduced graphene oxide/Co–BDC MOF composite (Co–BDC/rGO) by a hydrothermal method and subsequently pyrolyzed it to obtain a reduced graphene oxide/MOF-derived nanoporous carbon hybrid (NPC/rGO). Notably, in contrast to conventional MOF/rGO hybrids in which the MOF phase is directly used as the electroactive component, the Co–BDC framework, lamellar [Co_2_(OH)_2_(1,4-BDC)]*_n_*, was intentionally employed here as a precursor to construct the final electrode architecture after carbonization. In this study, rGO was used as a support for the growth of Co–BDC: the oxygen-containing functional groups on basal planes and edges of rGO nanosheets provide anchoring sites for Co^2+^, enabling the formation of an integrated Co–BDC framework on the graphene surface. After annealing, the rGO sheets retained within the hybrid act as an efficient nanoscale current collector and long-range charge-transport network and, importantly, as a morphological/structural template that guides the MOF-to-carbon conversion, yielding a more integrated porous carbon architecture (NPC/rGO). To further substantiate the stability advantage of this design, cobalt dissolution into the electrolyte was quantitatively assessed after electrochemical testing, revealing markedly reduced Co leaching for rGO-containing electrodes compared with the rGO-free counterpart, thereby directly linking the hybrid architecture to chemical stability. Finally, a high-performance asymmetric supercapacitor device (NPC/rGO-10//rGO) was successfully assembled to evaluate the practical applicability of the developed electrode.

## 2 Experimental Section

### 2.1 Materials

All of the reagents were of analytical grade, obtained from Merck or Sigma-Aldrich, and used without further purification.

### 2.2 Synthesis of Co-BDC MOF and Co-BDC/rGO Hybrid Nanocomposite

First, graphite oxide (GO) was prepared from commercial graphite powder using a modified Hummers’ method described in the literature.^35^ Anal. Exp. for rGO: O, 47.1%; C, 31.2%; H, 19.6%; and N, 2.1%. The elemental analyses showed that rGO is composed of 47.1% O, 31.2% C, 19.6% H, and 2.1% N. Then, the GO powder obtained was thermally reduced at 300 °C under a nitrogen atmosphere, following a methodology adapted from the work of Botas et al.^35^ The reduced material was named the rGO.

The synthesis of Co-BDC MOF was adapted from the procedure reported by Marso et al.^36^ and Narciso et al.^37^ The Co-BDC MOF was prepared from 5.8 g of Co­(NO_3_)_2_·6H_2_O dissolved in 5 mL of distilled water, and approximately 5.0 g of terephthalic acid was dissolved in 20 mL of dimethylformamide (DMF). The acid solution was slowly added to the cobalt salt solution. The resultant mixture was heated at 150 °C for 72 h in a Teflon-lined stainless-steel autoclave. The light pink solid obtained was washed with a 0.1 mol L^–1^ sodium hydroxide solution and distilled water. After, the product was washed with methanol and dichloromethane three times and dried in an oven at 150 °C for 72 h. Co-BDC/rGO hybrid nanocomposites were synthesized similarly to the synthesis of Co-BDC MOF. The Co-BDC/rGO composites were prepared in two different concentrations. The cobalt salt solution was mixed with a terephthalic acid solution and added to a dispersion of 10 mg or 20 mg of reduced graphene oxide (rGO) in 10 mL of DMF. The resultant dispersion was heated at 150 °C for 72 h in a Teflon-lined stainless-steel autoclave. The dark-brown solid obtained was washed with methanol and dichloromethane three times and dried in an oven at 150 °C for 72 h. The Co-BDC/rGO hybrid nanocomposites prepared with 10 and 20 mg of GO were named Co-BDC/rGO-10 and Co-BDC/rGO-20, respectively.

### 2.3 Preparation of MOF-Derived Nanoporous Carbon

The pyrolysis process was carried out using a quartz tube furnace. The as-obtained sample powders (Co-BDC MOF, Co-BDC/rGO-10, and Co-BDC/rGO-20) were heated to 800 °C at a heating rate of 10 °C min^–1^ under a N_2_ flow for 5 h. After cooling down to room temperature, the black powder was collected and labeled as NPC, NPC/rGO-10, and NPC/rGO-20, respectively, to the initial samples Co-BDC, Co-BDC/rGO-10, and Co-BDC/rGO-20. For comparison, individually annealed GO was also synthesized using the same procedure, yielding a pure rGO sample without any Co-BDC MOF. This rGO material was subsequently used as a control electrode and processed according to the same electrode fabrication protocol and electrochemical testing conditions used for the composite electrodes, allowing direct assessment of the role of the MOF-derived carbon component. Scheme 1 illustrates the strategy employed to fabricate the Co-BDC/rGO hybrid nanocomposite.

**Scheme 1:**
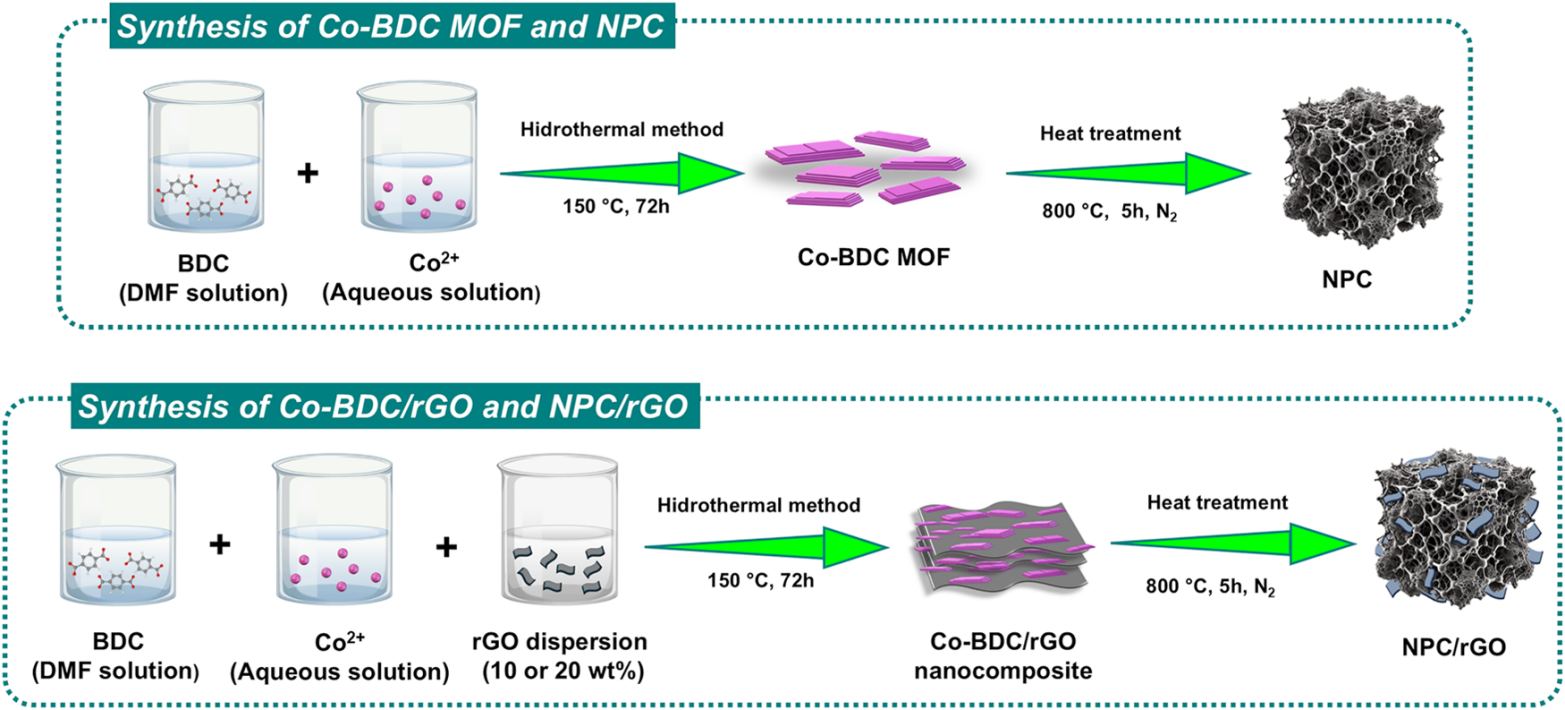
Schematic Showing the Preparation Procedure of MOF-Derived Nanoporous Carbon

### 2.4 Materials Characterization

The materials obtained in this work were characterized using various techniques. Powder X-ray diffraction patterns (DRX) were obtained with an X-ray Diffraction System Model D8-Discover Bruker using Cu-kα radiation (λ = 1.5418 Å), working at 40 kV and 40 mA. Intervals of 0.05° with a counting time of 1.0 s in the 2θ range of 5–80 degrees were used. Fourier Transformed Infrared (FTIR) spectra were recorded on a Shimadzu IRPrestige-21 spectrophotometer, ranging from 4000 to 400 cm^–1^. Thermogravimetric analysis (TGA) was performed under a synthetic air atmosphere (25 mL min^–1^) and heated from room temperature to 800 °C, at a heating rate of 5 °C min^–1^ on a TA Instruments TGA Q5000. To assess chemical stability under electrochemical operation, cobalt dissolution into the electrolyte was quantified by Inductively Coupled Plasma Mass Spectrometry (ICP–MS) after electrochemical measurements. This analysis was performed on electrolytes collected from cells cycled with the carbonized electrodes (NPC and NPC/rGO), thereby probing Co retention in the operational MOF-derived carbon architecture rather than in the pristine MOF precursor. Scanning electron microscopy (SEM) images were collected on a Quanta 200 FEG-FEI. The samples were coated with gold (5 nm) before the analysis. Elemental analyses of the samples were conducted using an energy-dispersive X-ray spectrometer (EDX) attached to the SEM. Transmission electron microscopy (TEM) was performed using a FEI TECNAI G2 electron microscope equipped with a thermionic gun and operated at an accelerating voltage of 200 kV. The sample was prepared by dispersing the material in acetone using a low-power ultrasonic bath, followed by deposition onto copper grids and solvent evaporation in air at room temperature. In addition to qualitative morphological analysis, a quantitative evaluation of the cobalt nanoparticle size distribution was carried out based on representative TEM micrographs. A total of 49 individual Co nanoparticles that could be clearly resolved were selected, avoiding regions affected by particle overlaps or insufficient contrast within the porous carbon matrix. The particle size was determined using the ImageJ software by measuring the projected size of each nanoparticle, and the resulting data were used to construct the particle size distribution and estimate the average nanoparticle size. Raman spectra were recorded by a Senterra from Bruker instrument equipped with an Olympus BX51 optical integrated microscope A He/Ne laser operating at 633 nm, and 10 mW power, 10 s, and 10 coadditions were used as the exciting source.

### 2.5 Electrochemical Testing

The working electrode was prepared by mixing the active materials (NPC, NPC/rGO-10, NPC/rGO-20, and rGO), multiwalled carbon nanotubes (MWCNT, Nanocyl NC 7000), and polyvinylidenedifluoride (PVDF) in a 90:5:5 mass ratio, using 1-methyl-2- pyrrolidone (NMP) as a solvent. The mixture was subjected to a low-power ultrasound bath for 40 min and subsequently stirred for 12 h (350 rpm). After that, the resulting slurry was coated onto a Ni foam current collector with a mass loading of ∼1,2 mg cm^–2^, which was dried at 90 °C for at least 2 h to remove NMP before the electrochemical test.

Electrochemical measurements were carried out using an electrochemical workstation (Biologic EC-Lab SP-300 and multichannel CS 2350 Corrtest) in a three-electrode system and a two-electrode asymmetric supercapacitor system at 25 °C. In all cases, a “T-type” Swagelok cell was used with glass fiber as separators. A glassy microfiber filter (Whatman) was used as a separator. The electrochemical measurements in a 3 M KOH aqueous electrolyte were performed using the Swagelok cell with Au as the counter electrode and Ag|AgCl|KCl­(3.5 M) as the reference electrode. The electrochemical behavior of the samples was evaluated by cyclic voltammetry (CV), galvanostatic charge–discharge (GCD), and electrochemical impedance spectroscopy (EIS) tests. The CV curves were performed under a potential window from 0 to 0.6 V at different scan rates in the range from 5 to 100 mV s^–1^.

Galvanostatic charge–discharge measurements were investigated in the potential window from 0 to 0.55 V at different current densities of 1 to 4.5 A g^–1^. EIS was taken in the frequency range from 100 kHz to 1 mHz with an amplitude of 10 mV. EIS experiments (10 mV; AC current perturbation) were performed at the open-circuit potential.

All calculations of electrochemical properties, such as specific cell capacitance and coulomb efficiency (ε), were calculated according to equations from the literature, as in the Supporting Information. The capacitance values are normalized to the total mass of the electrode excluding the polymer binder (PVDF). For the assembled two-electrode device, all reported specific capacitance values were calculated by normalizing the measured cell capacitance to the total mass of active materials in both electrodes (i.e., the combined mass of the positive and negative electrodes), in accordance with established methodologies for asymmetric supercapacitor devices.^38,39^

## 3 Results and Discussion

### 3.1 Material Morphology and Structural Characterization

To confirm the proper formation of the Co-BDC MOF structure and its composites Co-BDC/rGO-10 and Co-BDC/rGO-20, powder X-ray diffraction measurements were carried out as shown in Figure 1a. The MOF and the crystalline phases observed in the composites correspond to the known structure of [Co_2_(OH)_2_(1,4-BDC)]_n_, which crystallizes in the monoclinic system with space group *C*2/*m*.^40^ The experimental diffraction patterns were compared with a simulated reference pattern generated from crystallographic data obtained from the Cambridge Crystallographic Data Centre (CCDC, Refcode QASLIF, CCDC #153067), allowing the identification of the main crystallographic planes. Moreover, the patterns observed for the composites closely match those of the pristine Co-BDC MOF, indicating that the crystalline structure is preserved and not disrupted by the presence of rGO during synthesis. The average crystallite sizes (*d̅*) were also estimated from the PXRD patterns using the Scherrer equation, applied to the most intense diffraction peak at 2θ = 15.77° corresponding to the (400) plane. The resulting values confirm the formation of nanometric particles, with *d̅* values of 30.2, 26.7, and 27.6 nm for the Co-BDC MOF, Co-BDC/rGO-10, and Co-BDC/rGO-20 samples, respectively. Notably, the diffraction patterns of Co-BDC/rGO closely match those of pristine Co-BDC, indicating that rGO does not disrupt MOF crystallization but provides a substrate for in-situ growth. This precursor-level structural preservation is critical for obtaining an integrated MOF/rGO architecture prior to carbonization.

**Figure 1:**
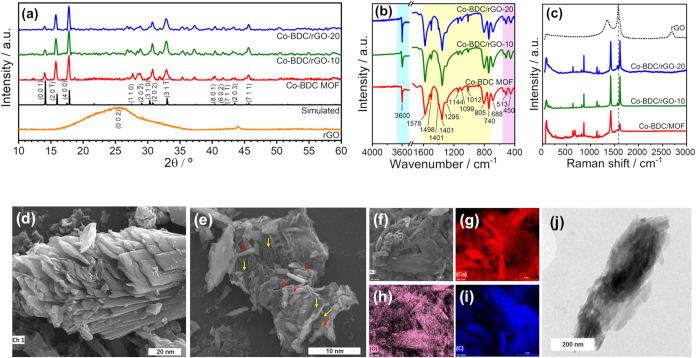
XRD patterns (a), FTIR spectrum (b), and Raman spectra (c) of the prepared Co-BDC MOF, Co-BDC/rGO-10, Co-BDC/rGO-20, and rGO, respectively. SEM images of Co-BDC MOF (d) and Co-BDC/rGO-10 (e). EDX elemental mapping of Co-BDC/rGO-10 (f–i, Co: red, O: pink, and C: blue). TEM image of Co-BDC/rGO-10 (j).

For completeness, the X-ray diffraction (XRD) pattern of reduced graphene oxide (rGO) is included in Figure 1a. The rGO sample exhibits a broad diffraction feature centered at approximately 24–26° (2θ), which is assigned to the (002) plane of graphitic carbon.^41,42^ The broad and low-intensity nature of this reflection is characteristic of rGO and indicates a low degree of graphitic ordering, a loss of long-range stacking order between graphene layers, and partial restoration of the sp^2^ carbon framework after reduction. In the Co-BDC/rGO composite materials, the (002) reflection of rGO is not clearly resolved due to its low mass fraction in the composites and the dominance of more intense diffraction peaks associated with the Co-BDC-derived phases, which effectively suppress the contribution of the rGO signal.

Vibrational spectroscopy was also employed to characterize the materials. All active and intense modes observed in the FTIR and Raman spectra of the pristine Co-BDC MOF were also detected in the composites. Figure 1b presents the FTIR spectra, showing the μ–OH bridging hydroxyl stretching vibration at 3600 cm^–1^ (ν­(OH)), as well as the symmetric (ν_s_(COO^–^)) and asymmetric (ν_as_(COO^–^)) stretching modes of carboxylate groups coordinated to Co^2+^, located at 1356 and 1498 cm^–1^, respectively. The difference between these wavenumbers (Δν < 200 cm^–1^) also indicates a bridging coordination mode, where the two oxygen atoms are bonded to different cobalt centers.^43^ Several additional medium-to-weak bands are present between 1600 and 700 cm^–1^, corresponding to the stretching vibrations of the aromatic ring (C = C) as well as the stretching and in-plane bending modes of C–H bonds from the benzene ring of the BDC linker. The bands related to Co–O bonding, corresponding to bending (δ­(Co–O)) and stretching (ν­(Co–O)) vibrations, appear at lower wavenumbers and with low intensity, specifically at 513 and 450 cm^–1^.^44,45^

Raman spectra, which are more sensitive to symmetric modes and aromatic ring vibrations, are shown in Figure 1c. The most intense signals at 1610 and 1420 cm^–1^, along with a weaker band at 1135 cm^–1^, are associated with in-plane vibrations of the benzene ring. The modes assigned to ν­(Co–O) and δ­(O–Co–O) within the CoO_6_ octahedron occur between 450 and 600 cm^–1^, but due to their low intensity, they could not be distinguished from baseline noise in this work. Additionally, in the composites, the G-band at 1575 cm^–1^—related to the vibration of sp^2^-hybridized carbon in two-dimensional graphite-like domains—and the D-band at 1340 cm^–1^—associated with structural defects in carbon materials—are almost entirely suppressed.

Scanning electron microscopy (SEM) and transmission electron microscopy (TEM) were employed to investigate the microstructures of the samples. SEM images (Figure 1d) show Co-BDC MOF with a typical 2D lamellar morphology, typical of some MOFs, with large stacked layers forming a porous network.^32^ Upon rGO incorporation (Figure 1e), the MOF retained its lamellar structure, not showing morphological alteration, as highlighted in red. After the introduction of rGO, the MOF particle grows *in situ* on the layered structure of the rGO substrates (highlighted in yellow) in the Co-BDC/rGO hybrid nanocomposite. The MOF, a closely packed lamellar, covered almost the entire area of the rGO, including both the surface and the edge. Additionally, the incorporation of rGO reduced the size of the Co-BDC particles and led to regions of rGO agglomeration. To analyze the spatial distribution of Co, C, and O in the Co-BDC/rGO hybrid nanocomposite, EDX elemental mapping of the material was performed (Figure 1f–i). The results reveal that Co, C, and O are homogeneously distributed, indicating a strong integration of the metal–organic framework with carbon materials. The TEM image of Co-BDC/rGO-10 (Figure 1j) reveals the integration of rGO with the Co-BDC MOF. The Co-BDC MOF is uniformly distributed on both sides of the rGO sheets, forming well-defined, blade-like nanostructures along the edges of the hybrid.

Upon pyrolysis of the Co-BDC MOF and its rGO-containing composites under an inert atmosphere, nanostructured porous carbons with a hierarchical architecture are formed, which are desirable for supercapacitor applications. The pyrolysis of the organic ligand (BDC) releases gases such as CO, CO_2_, and H_2_, while also generating solid carbon that acts as a reducing agent.^46^ As a result, Co^2+^ ions originally present in the MOF can be partially converted to CoO and subsequently reduced to metallic cobalt (Co^0^), according to the following reaction

1 CoO(s)+C(s)→Co(s)+CO(g)

The formation of metallic Co^0^ was confirmed. Figure 2a shows the PXRD pattern obtained after pyrolysis of the Co-BDC/rGO-10 composite, resulting in the nanoporous carbon composite denoted as NPC/rGO-10. The observed diffraction peaks corresponding to the (111), (200), and (220) planes are attributed to metallic cobalt with a face-centered cubic (FCC) structure, typically indexed in the *Fm*3̅*m* space group, which is characteristic of a close-packed lattice (JCPDS 00–001–1255). The average crystallite size was estimated to be 22.8 nm based on the (111) reflection. Additionally, a broad diffraction halo centered around 25° is observed, which is associated with mostly amorphous carbon derived from the decomposition of the MOF structure.

**Figure 2:**
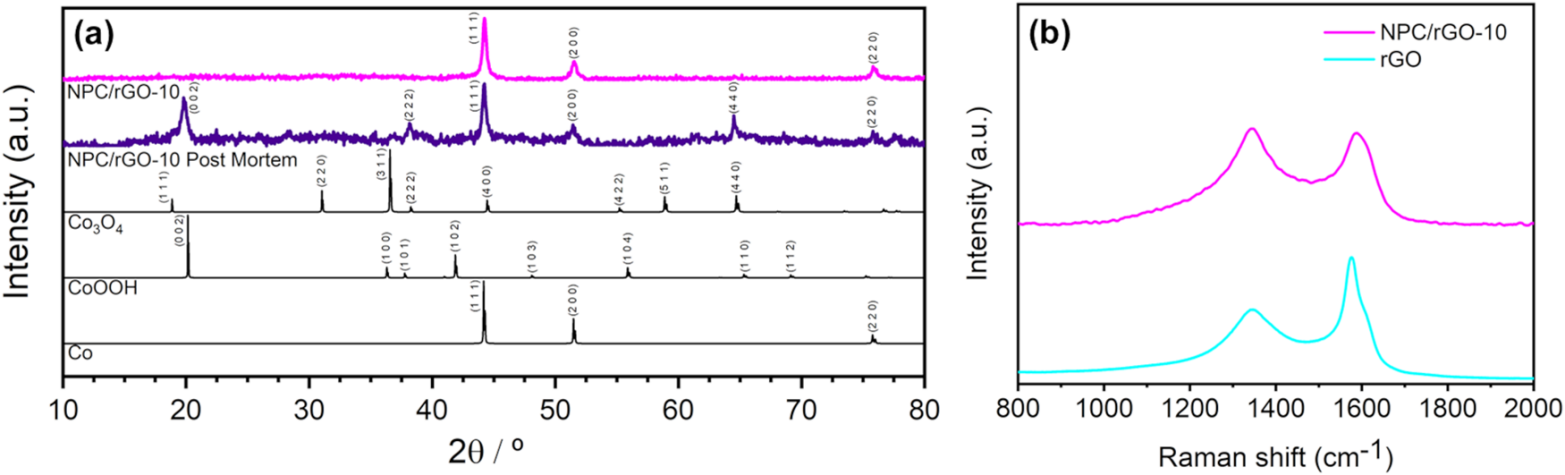
XRD patterns of the as-prepared NPC/rGO-10 and the corresponding post-mortem electrode after prolonged charge–discharge cycling (a). Raman spectra of NPC/rGO-10 and pristine rGO (b).

Raman spectroscopy was employed to assess the extent of graphitization in the carbonized samples. As illustrated in Figure 2b, the spectra corresponding to rGO and NPC/rGO-10 are presented. The characteristic bands of carbon materials, corresponding to the D-band (disordered carbon) at 1345 cm^–1^ and the G-band (graphitic carbon) at 1580 cm^–1^, were clearly observed. The structural disorder and degree of graphitization were quantitatively assessed by evaluating the intensity ratio (*I*_D_/*I*_G_) between the D and G bands. The calculated *I*_D_/*I*_G_ values for rGO and NPC/rGO-10 were 0.89 and 1.01, indicating a progressive increase in the defect density. The higher *I*_D_/*I*_G_ ratio observed for NPC/rGO-10 is attributed to the increased density of structural defects induced by the incorporation of Co-BDC MOF during the graphitization process.^47,48^ Despite the potential decrease in electrical conductivity that may result from this effect, it is widely recognized that a higher defect concentration enhances electrochemical reactivity and accelerates the redox kinetics of carbon-based materials in energy-storage and conversion applications.^47^

Figure 3a–f presents SEM micrographs of the material obtained from the thermal annealing of Co-BDC MOF in the absence of rGO. The images reveal Co-doped nanoporous carbon particles with irregular morphologies and heterogeneous size distributions, indicating the formation of a structurally diverse porous framework. This reflects the absence of structural support from rGO during the thermal treatment. Conversely, the thermally treated hybrid composite (NPC/rGO-10) exhibited a more controlled thermal transformation (Figure 3g–l). The rGO acted as a stabilizing matrix, forming particles with more uniform shapes and sizes. This effect can be attributed to the strong interaction between the oxygenated functional groups of rGO and the metal ions. Thus, the metal–organic framework can be easily attached to both sides of rGO during the reaction protocol, promoting the formation and stabilization of intermediate products on the rGO surface.^49,50^ The NPC/rGO-10 exhibited a highly dense and interconnected porous architecture (as highlighted in Figure 3g,h), which is highly advantageous for providing an accessible surface for fast ion transport in the supercapacitor. In the case of NPC/rGO-10, the distribution of Co, O, and C was homogeneous due to the decomposition of MOF particles and their subsequent reorganization onto the rGO sheets (Figure 3i–l).

**Figure 3:**
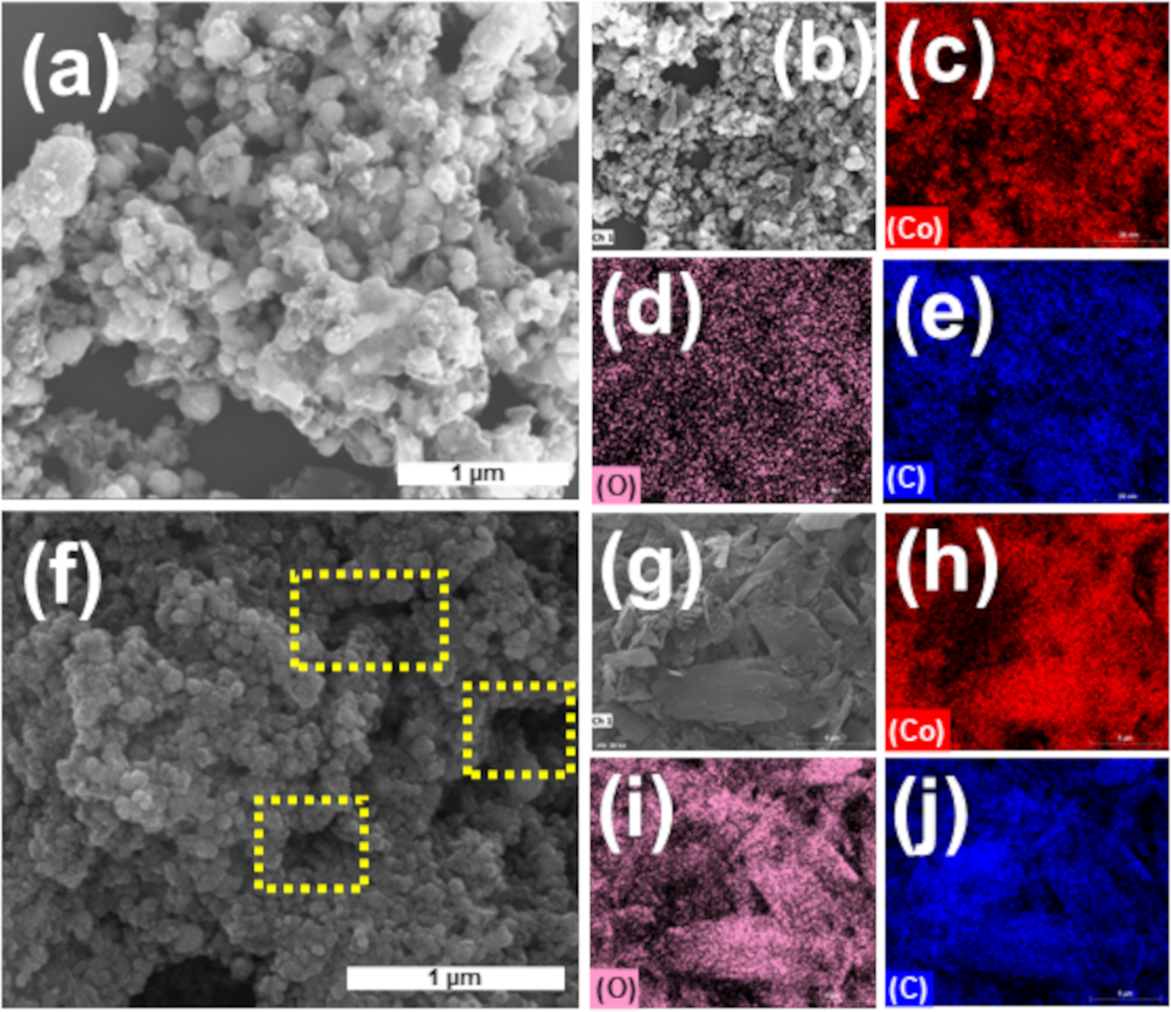
SEM images of NPC (a, b) with corresponding EDX elemental mapping (c–e), NPC/rGO-10 (f, g) with corresponding EDX elemental mapping (h–j) (Co: red, O: pink, and C: blue). Highlight: in (f), a porous architecture accessible to electrolyte ions is observed.

The elevated temperature during thermal treatment converts the MOF to porous carbon particles and facilitates a greater reduction of rGO. TEM images of NPC/rGO-10 are presented in Figure 4. The NPC/rGO-10 displayed a well-developed porous framework, which can promote rapid ion-diffusion and effective electrolyte accommodation (Figure 4a,b). After pyrolysis, structures with crystalline domains are formed, as highlighted in Figure 4c. The unique structure of NPC/rGO-10 satisfies several key criteria essential for an optimal supercapacitor electrode. Figure 4d reveals the presence of Co^0^ nanoparticles with an almost spherical morphology, uniformly dispersed, and attached to rGO sheets. TEM-based particle size analysis reveals a homogeneous distribution of cobalt nanoparticles embedded in the MOF-derived porous carbon framework, with particle diameters spanning the 15–69 nm range (Figure S1, Supporting Information).

**Figure 4:**
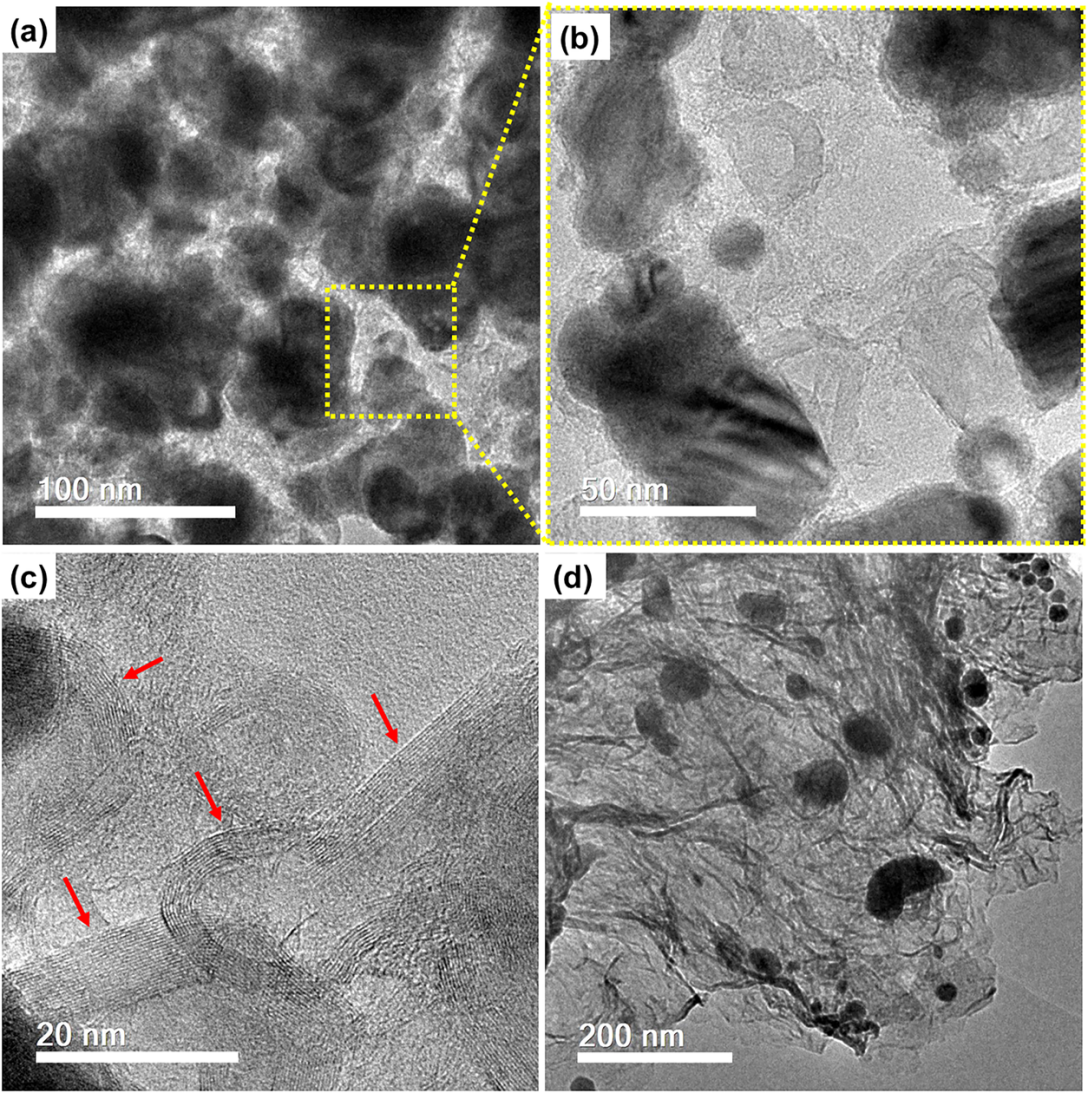
TEM images of NPC/rGO-10. A hierarchically carbon structure accessible to electrolyte ions is presented and highlighted in (a,b), and a high-resolution image of NPC/rGO-10 (c) and Co^0^ nanoparticles attached on rGO (d).

### 3.2 Electrochemical Performance of the Electrodes

Electrochemical tests were conducted in NPC, NPC/rGO-10, NPC/rGO-20, and rGO electrodes to determine their suitability as electrode materials for supercapacitors. First, standard three-electrode systems were performed using a voltage window of 0 to 0.60 V in 3 M KOH electrolyte. Figure 5a shows typical CV curves of diverse electrode materials over the potential range 0.0–0.60 V (vs Ag|AgCl) at a scan rate of 5 mV s^–1^.

**Figure 5:**
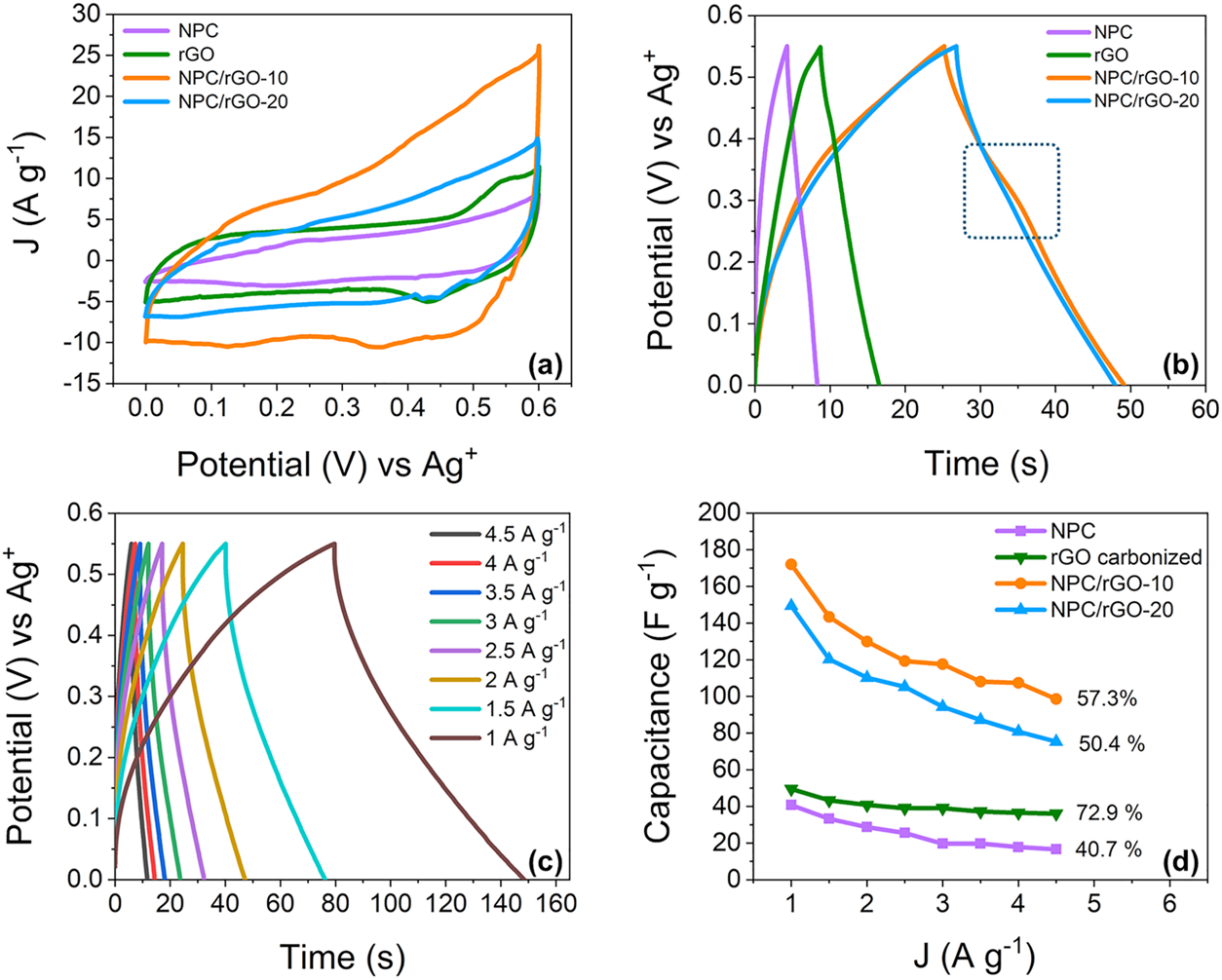
Electrochemical characterization of NPC, rGO, NPC/rGO-10, and NPC/rGO-20 in a 3-electrode cell: CV plots at a scan rate of 5 mV s^–1^ (a) and GCD plots at 3 A g^–1^ (b). GCD curves of NPC/rGO-10 at different current densities (c) and variation of specific capacitance with current density (d).

The CV area of NPC/rGO-10 (Figure 5a) is significantly larger than that of the other tested samples, indicating that NPC/rGO-10 possesses a much higher specific capacitance and improved electrochemical activity. All samples exhibit distorted voltammograms compared to a typical EDLC behavior, indicating a combination of double-layer capacitor and pseudocapacitor characteristics. The incorporation of rGO during the synthesis of NPC/rGO-10 appears to have contributed to its enhanced conductivity, as demonstrated by its superior current response, highlighting the material’s efficiency in charge storage and transfer.^17^ The absence of well-defined redox peaks in the cyclic voltammetry profiles can be attributed to the nanostructured nature of metallic cobalt. As evidenced by the crystallite sizes estimated from XRD data using the Scherrer equation (∼20 nm), the highly dispersed Co^0^ species provide a large number of electroactive sites. Such a nanostructured configuration minimizes mass-transport limitations, enabling fast, reversible faradaic reactions over a broad potential range and thereby smoothing out distinct redox features in the CV curves. Additionally, the oxidation and reduction peaks are more intense in the rGO electrode, which is composed exclusively of rGO, probably due to the residual oxygenated functional groups that remained in the graphitic structure even after the thermal reduction process.^38^ CV curves for all samples at scan rates of 5 and 100 mV s^–1^ are shown in Figure S2 (Supporting Information).

In order to better understand the electron-transfer processes and to determine the redox mechanisms of the system, the dependence of the peak current on the scan rate was analyzed (Figure S3, Supporting Information). All electrodes exhibited b-values (slope) in the range 0.56–0.72, indicating that charge storage is governed by a mixed mechanism involving both diffusion-controlled ion transport and adsorption-controlled processes. The pristine NPC electrode exhibits a b-value close to 0.5, characteristic of diffusion-limited behavior, suggesting that ion transport within its porous framework plays a dominant role in the charge storage process. In contrast, incorporating rGO results in progressively higher b-values, reflecting an increased contribution from adsorption-controlled charge storage. This behavior can be attributed to the enhanced electronic conductivity and the electrochemically accessible area provided by the rGO network, which facilitates fast redox reactions of species confined to or near the electrode surface.

In alkaline electrolytes, the faradaic behavior of cobalt-based electrodes is governed by a series of well-established redox transitions.^51,52^ These reversible redox reactions contribute significantly to the pseudocapacitive charge storage process, enabling rapid electron transfer and enhancing the overall electrochemical performance of cobalt-containing electrodes in supercapacitor applications. Initially, metallic cobalt enhanced oxidation to form Co­(OH)_2_ according to the following reactions

2 $$\mathrm{Co}+2{\mathrm{OH}}^{-}⇌\mathrm{Co}{\left(\mathrm{OH}\right)}_{2}+2e^{-}$$

3 $$3\mathrm{Co}{\left(\mathrm{OH}\right)}_{2}+2{\mathrm{OH}}^{-}⇌{\mathrm{Co}}_{3}O_{4}+4H_{2}O+2e^{-}$$

4 $${\mathrm{Co}}_{3}O_{4}+{\mathrm{OH}}^{-}+H_{2}O⇌3\mathrm{CoOOH}+e^{-}$$

5 $$\mathrm{CoOOH}+{\mathrm{OH}}^{-}⇌{\mathrm{CoO}}_{2}+H_{2}O+e^{-}$$

To investigate the chemical state of cobalt after electrochemical operation, post-mortem X-ray diffraction (XRD) analysis was performed on the cycled NPC/rGO-10 electrode and compared with the pristine material (Figure 2a). The pristine electrode exhibits diffraction features characteristic of metallic Co^0^, consistent with the high-temperature pyrolysis under an inert atmosphere. After prolonged cycles, the maintenance of peaks related to metallic cobalt and additional peaks related to cobalt oxyhydroxide (CoOOH) are observed, in addition to diffraction peaks of cobalt oxide (Co_3_O_4_) appearing to a lesser extent. The appearance of oxidized oxyhydroxide–oxide phases indicates the partial oxidation of cobalt species during electrochemical operation. These observations are consistent with the participation of cobalt species in the pseudocapacitive charge storage processes.

The galvanostatic charge–discharge measurements were conducted to evaluate the capacitive performance. Figure 5b presents the corresponding GCD curves for NPC, NPC/rGO-10, NPC/rGO-20, and rGO at a current density of 3 A g^–1^. The results indicate that composites containing rGO exhibit significantly longer discharge times and higher Coulombic efficiency than pure NPC and rGO. Specifically, the specific capacitance values of NPC/rGO-10, NPC/rGO-20, NPC, and rGO are 172.0, 150.4, 40.8, and 49.5 F g^–1^, respectively, at a current density of 1.0 A g^–1^. The synergistic interaction between rGO and MOF-derived nanoporous carbon plays a crucial role in enhancing electrochemical performance, because without rGO, the capacitive performance of the nanoporous carbon is poor, and rGO by itself does not significantly contribute to electric double-layer capacitance. Furthermore, it can be noted that the NPC/rGO-10 composite exhibits a minor distortion in the GCD curve (observed at approximately 0.3 V), characterized by a slight curvature that suggests a faradaic contribution, consistent with the observations from the CV curves. The charge–discharge curves of the NPC/rGO-10 composite at all investigated current densities are presented in Figure 5c. Similar figures are shown in Figure S4 (Supporting Information) for the other materials, evaluated under the same conditions.

The specific capacitance of each sample as a function of the current density is plotted in Figure 5d. An increase in current density results in a decrease in specific capacitance, which can be attributed to diffusion limitations at the electrode surface.^53^ The NPC/rGO-10 electrode provided the highest capacitance of 172.0 F g^–1^ at a current density of 1 A g^–1^ and had a higher capacitance retention rate of ∼ 57%, probably due to the higher conductivity than other samples and its favorable morphological structure.^54^ Even at the highest current density that we tested (4.5 A g^–1^), the specific capacitance of both hybrid nanocomposites (NPC/rGO-10 and NPC/rGO-20) is higher than that of the samples (NPC and rGO) tested at lower current densities. By incorporating graphene to create a porous interconnected structure, the electron transport capability is enhanced, the ion-diffusion and transport pathways are shortened, and the capacitive performance is improved.^17^ Furthermore, ICP-MS was employed to analyze the Co^2+^ concentration in the electrolyte collected after multiple charge–discharge cycles, cyclic voltammetry, and electrochemical impedance spectroscopy measurements. The NPC/rGO-10 and NPC/rGO-20 electrodes exhibited a lower cobalt concentration in the electrolyte (0.007 mg L^–1^) compared to that of the NPC electrode without rGO (0.020 mg L^–1^). These results provide evidence consistent with reduced cobalt leaching in the presence of rGO. It appears that these nanosheets help to keep the Co trapped in the structure of the hybrid nanocomposite, contributing to the faradaic and electric double-layer processes in the electrode. Additionally, with the addition of graphene to form a porous interconnected structure, the electron transport capability is improved, the diffusion and transport distance of ions are shortened, and the capacitive performance is enhanced.

The resistances associated with charge-transfer processes were investigated by electrochemical impedance spectroscopy around the open-circuit potential, as evidenced by the Nyquist plots in Figure 6a. The impedance response was fitted using the equivalent circuit as shown in highlight in Figure 6a, comprising the series resistance (*R*_s_)—which accounts for the resistive contributions from the electrolyte, the electrode, and the electrode–electrolyte contact—the charge-transfer resistance (Rct), a constant phase element (*Q*_1_) describing the nonideal capacitive behavior at the electrode–electrolyte interface, as well as additional elements associated with ion-diffusion processes.^55,56^

**Figure 6:**
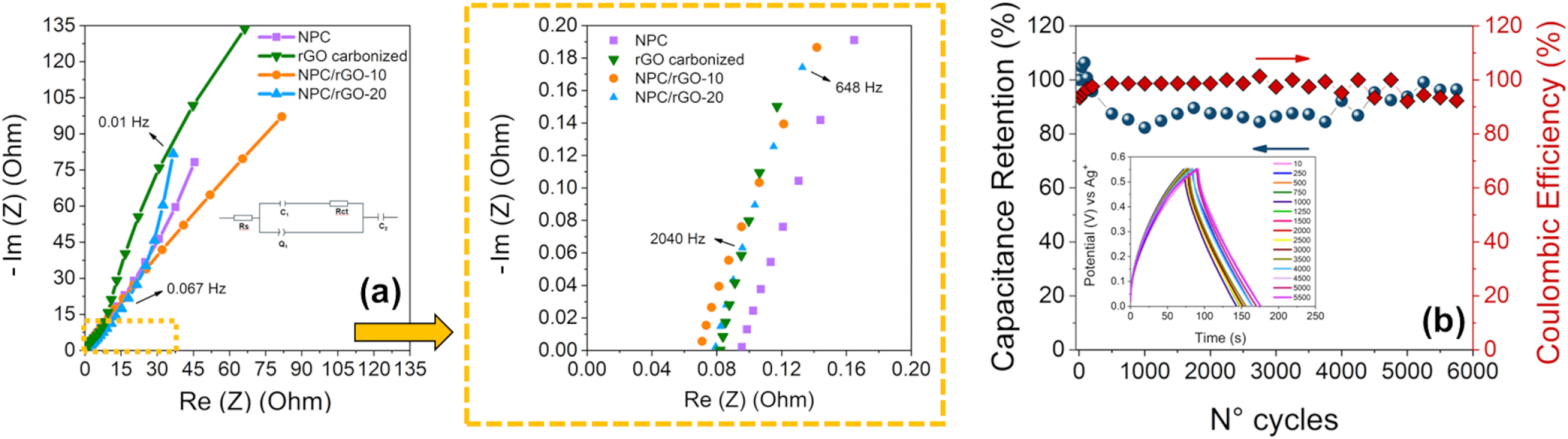
Nyquist plots of electrodes prepared. The inset shows the curves in the low-frequency region (a). Cyclic stability measurements for NPC/rGO-10 are evaluated at 3.5 A g^–1^ over 6,000 charge/discharge cycles, and the Coulombic efficiency values over the cycles are also shown (right axis) (b).

In the high-frequency domain (inset of Figure 6a), the intercept on the real axis corresponds to the overall series resistance (*R*_s_). Notably, the *R*_s_ values were very close for all electrodes, varying only from 0.07 to 0.10 Ω. Specifically, NPC/rGO-10 and NPC/rGO-20 exhibited slightly lower *R*_s_ values (0.07 and 0.08 Ω, respectively) than rGO (0.09 Ω) and NPC (0.10 Ω). Although these differences are minor, they suggest marginally improved electrolyte permeation and reduced ionic transport resistance in the rGO-containing composites, which can be attributed to the formation of continuous conductive pathways and enhanced wettability within the porous architecture. The charge-transfer resistance (Rct), extracted from the semicircle diameter in the medium-frequency region, exhibits marked variations among the electrodes. The rGO electrode presents the lowest Rct value (20.1 Ω), implying faster charge-transfer kinetics, which can be rationalized by its high electronic conductivity and the effective accessibility of electroactive sites. Conversely, the NPC electrode shows the highest adduct (28.6 Ω), suggesting comparatively hindered charge-transfer processes, likely arising from the lower intrinsic conductivity of the porous carbon framework and less efficient interfacial electron transport. The NPC/rGO nanocomposites yield intermediate Rct values (23.5 Ω for NPC/rGO-10 and 25.0 Ω for NPC/rGO-20), demonstrating that incorporating rGO into the NPC matrix substantially improves charge-transfer kinetics relative to pristine NPC, owing to enhanced electronic conductivity and improved interparticle connectivity. Notably, increasing the rGO content from 10 to 20 wt % results in a modest increase in Rct, which may be associated with partial rGO sheet aggregation and/or blockage of active pores, thereby reducing the effectiveness of the electrode–electrolyte interface. In addition, the slope of the segment linear in the low-frequency region was associated with the Warburg impedance, primarily reflecting the ion transport resistance during the diffusion process.^57^

An essential metric for evaluating the performance of electrode materials is cycling stability, so Figure 6b shows the cycling stability of the electrode performed at a current density of 3.5 A g^–1^. The electrode NPC/rGO-10 showed excellent cycling performance, in which the capacitance was maintained at 96% after 6000 repetitive charge–discharge cycles, certifying the significantly superior energy-storage performance of the electrode during the repetitive charge–discharge cycle. Similar behavior was observed for the Coulombic efficiency, which was maintained at 92.3% throughout the cycling. The inset of Figure 6b shows that the charge/discharge curve profile remains practically constant over multiple cycles. The high stability of the composite electrode is attributed to the open pore structure, which allows fast transmission channels for electrons and ions, which improves the specific capacity and rate performance of the material.

The NPC/rGO-10 hybrid nanocomposite provides abundant electrochemically active sites for redox reactions, leading to a competitively high specific capacity. Its structure establishes efficient transmission channels for both electrons and ions, thereby enhancing the conductivity, structural stability, and overall electrochemical performance. Owing to the synergistic effect between the two components, NPC/rGO-10 as electrode materials revealed excellent electrochemical properties. The synergistic effect between the two components further contributes to the superior capacitive behavior of NPC/rGO-10, reinforcing its potential as a high-performance electrode material for energy-storage applications.

### 3.3 Electrochemical Performance of the Asymmetric Supercapacitor Device Based on NPC/rGO-10

To further evaluate the electrochemical performance of NPC/rGO-10 in practical applications, an asymmetric supercapacitor was fabricated using NPC/rGO-10 as the positive electrode, rGO as the negative electrode, and KOH 3 M as the electrolyte. This device is referred to as NPC/rGO-10//rGO ASC, and its schematic diagram is presented in Figure 7a. The CV curves of NPC/rGO-10//rGO ASC at various scan rates are shown in Figure 7b, which displays a quasirectangular area between 0 and 0.6 V. The CV curves retain their original shape throughout the variation in scan rate, indicating good rate capability. The GCD curves of the hybrid supercapacitor from 0.25 to 2 A g^–1^ are displayed in Figure 7c and exhibit a relatively high Coulombic efficiency of 95% across various current densities.^58^ The relationship between the current density and specific capacitance is presented in Figure 7d. The device exhibits high specific capacitance values of 27.3, 17.3, 9.7, 6.4, and 4.5 F g^–1^ at current densities of 0.25, 0.5, 1.0, 1.5, and 2 A g^–1^, respectively. The specific capacitance decreased as the current density increased, indicating diffusional restrictions.

**Figure 7:**
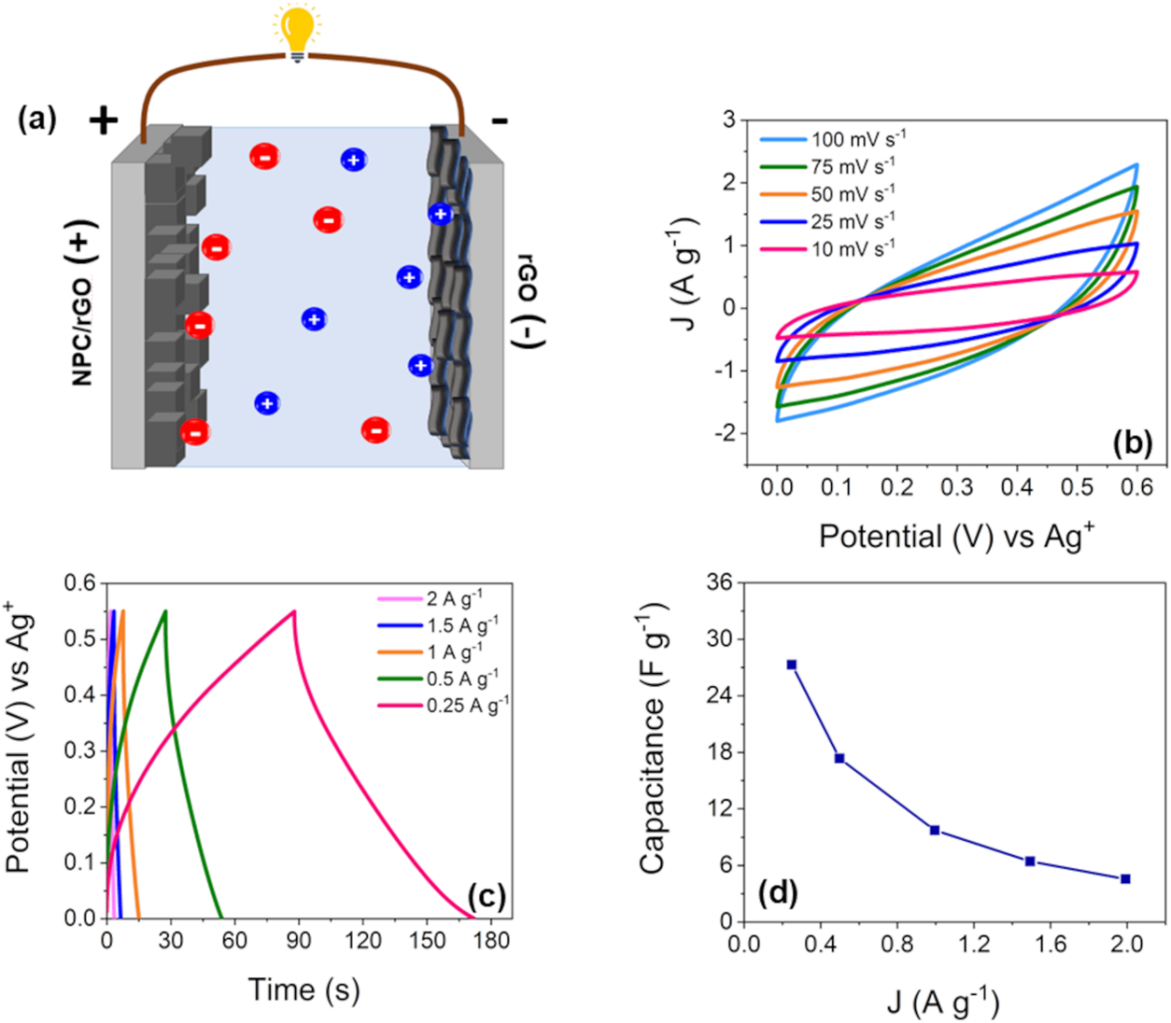
Schematic diagram of NPC/rGO-10//rGO ASC (a), cyclic voltammetry response at different scan rates (b), galvanostatic charge/discharge profile (c), and specific capacity versus current densities of NPC/rGO-10//rGO, respectively (d).

The cycling stability of the NPC/rGO-10//rGO ASC electrodes was evaluated through continuous charge/discharge cycles at a constant current density of 1 A g^–1^, as illustrated in Figure 8a. The inset presents the GCD profile of the NPC/rGO-10//rGO ASC, illustrating the charge–discharge behavior from cycles 1 to 5 and from cycles 29,995 to 30,000. The results demonstrate that the device retains 94.4% of its initial capacitance even after 30,000 cycles, demonstrating extraordinary stability when compared to those in previous studies such as Subramani et al.^59^ (92% retention observed after 5,000 cycles for CoS//AC HSC), Rakhi et al.^60^ (90% retained after 5,000 cycles for Co_9_S_8_ nanoflakes//AC), Dai et al.,^61^ (90% retention observed after 5000 cycles for Ni_3_S_2_/MWCNT-NC//AC), Singh et al.^62^ (92% retention observed after 1000 cycles for MWCNT/NiS/GNP based HSC), and Cheng et al.^63^ (88% retention observed after 5000 cycles for CuCoS_4_ NRAs//AC). Throughout the entire experiment, the cell’s Coulombic efficiency remains in the range of 93.5 to 100%, attesting to the cell’s stability (Figure 8a). This superior stability was confirmed by a CV, which was performed over 30,000 cycles. No pronounced variations were observed in the CV curve profiles, as shown in Figure 8b. The reversible capacity of NPC/rGO-10//rGO ASC electrodes subjected to varying current densities is illustrated in Figure 8c. As the current density increases, a gradual decline in the capacitance of the electrode material is observed. When the current density reaches 1.5 A g^–1^, the retention of capacitance remains at 63% after 240 cycles. Remarkably, upon returning the current density to 0.1 A g^–1^ after 270 cycles, the capacitance reaches the same values as the initial ones. This outstanding rate capability of the NPC/rGO-10//rGO ASC can be attributed to its open structure formed by the pyrolyzed MOF structures anchored on the rGO nanosheets, which facilitates rapid access to electrolyte ions and minimizes the ion-diffusion distance, as shown in Figure 8d. The complete-cell results further validate the proposed design principle, demonstrating that the rGO-integrated MOF-derived carbon architecture simultaneously provides efficient charge transport and structural robustness under prolonged cycling. The outstanding capacitance retention over 30,000 cycles evidence the operational stability of the carbonized Co-BDC/rGO-derived electrode, in agreement with the reduced cobalt dissolution quantified in this study. In addition, to evaluate the practical performance of NPC/rGO-10//rGO ASC, the energy density (*E*) and power density (*P*) were determined using Eqs (S5) and (S6), and the results obtained are displayed in the Ragone plot shown in Figure S5 (Supporting Information). Furthermore, a symmetric supercapacitor device prepared based on NPC/rGO-10//rGO allowed light from a 1.5 V red LED for 60 s, as highlighted in Figure 8c.

**Figure 8:**
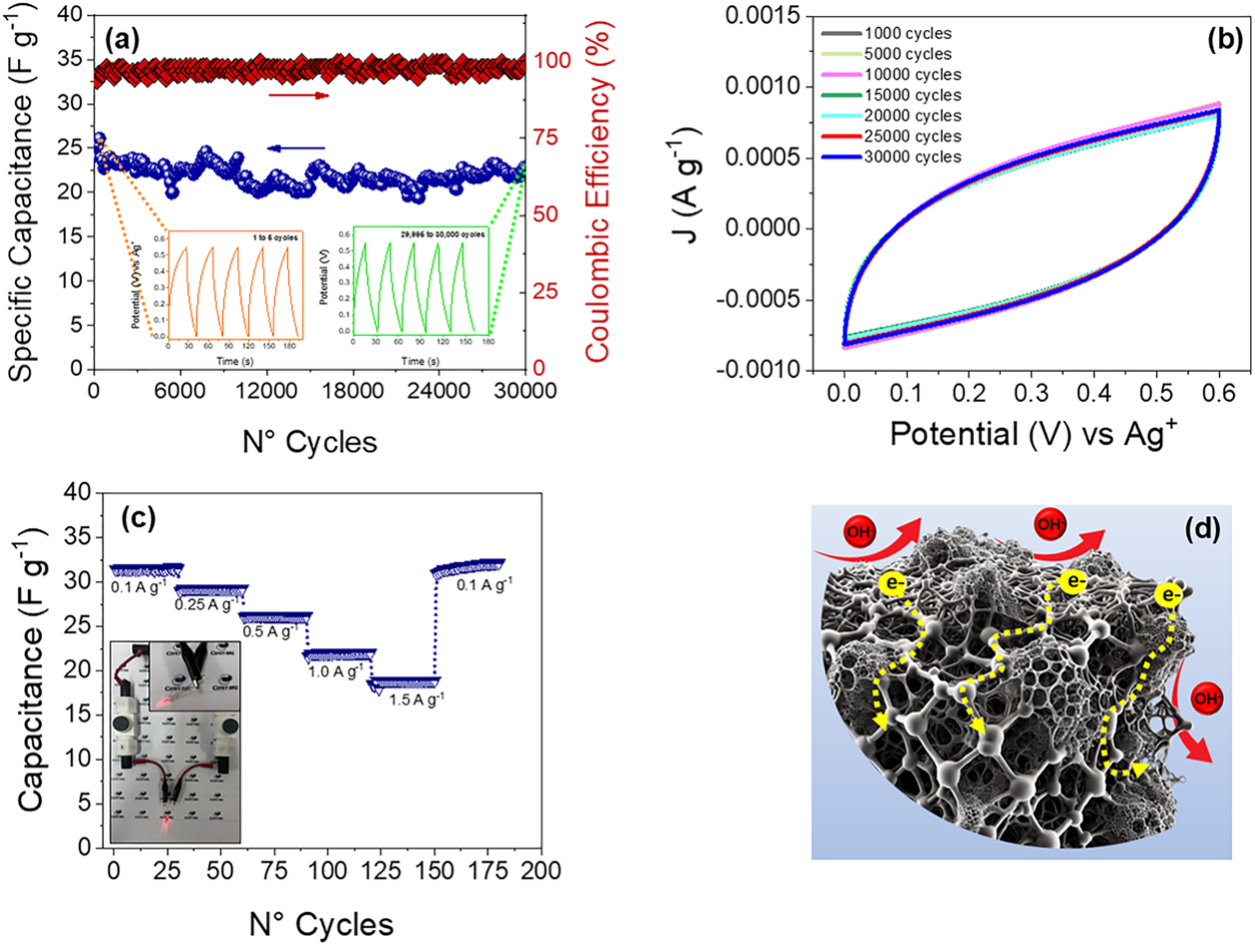
Cycling stability performance at 1 A g^–1^ with first 5 and the last 5 GCD cycles of NPC/rGO-10//rGO ASC device during the cycling stability test (a), profiles of cyclic voltammetry curves at 10 mv s^–1^ obtained over 30,000 cycles (b), reversible capacity of NPC/rGO-10//rGO ASC at various current densities ranging from 0.1 to 1.5 A g^–1^ (c), and schematic image of ion and charge transfer (d).

To further contextualize the electrochemical performance and highlight the relevance of the present system, a comparative analysis with representative literature reports is provided in Table S1 (Supporting Information). This comparison summarizes key performance metrics, including specific capacitance, energy density, power density, applied current density, operating voltage window, and cycling stability, for recently reported MOF-derived carbon- and graphene-based supercapacitors. As shown, the NPC/rGO-based ASC developed in this work delivers a competitive balance of energy and power densities together with electrochemical properties with excellent cycling, demonstrating that the combination of Co-BDC-derived porous carbon with rGO is an effective strategy for enhancing the electrochemical performance of asymmetric supercapacitors.

## 4 Conclusions

In this work, a Co-BDC MOF/rGO-derived nanohybrid was successfully synthesized and evaluated as an electrode material for supercapacitor applications. The in-situ incorporation of rGO during the MOF synthesis preserved the crystallinity of the Co-BDC framework and enabled the formation of an integrated precursor architecture prior to carbonization. Subsequent controlled pyrolysis resulted in a uniform and interconnected nanoporous carbon/rGO hybrid containing well-dispersed metallic Co^0^ nanoparticles. Among the investigated materials, the NPC/rGO-10 composite exhibited the most balanced electrochemical performance, combining a high specific capacitance, low internal resistance, and excellent cycling stability. The presence of metallic Co^0^ further contributed to the enhanced performance by enabling pseudocapacitive behavior through reversible redox transitions (Co^0^/Co^2+^ or Co^2+^/Co^3+^), particularly in aqueous electrolytes. From an originality perspective, this study demonstrates three key aspects: (i) a Co-BDC/rGO precursor strategy that preserves MOF crystallinity and enables intimate integration prior to carbonization; (ii) an rGO-assisted MOF-to-carbon transformation yielding a robust, interconnected nanoporous carbon/graphene architecture; and (iii) a quantitative evaluation of cobalt dissolution in the electrolyte, revealing cobalt concentrations in the electrolyte that are consistent with reduced leaching in rGO-containing electrodes under electrochemical operation. These structural and chemical features translate to enhanced electrochemical durability at both electrode and device levels. An asymmetric supercapacitor assembled using NPC/rGO-10 as the positive electrode and rGO as the negative electrode demonstrated stable operation over 30,000 charge–discharge cycles with minimal capacitance decay and sustained power output. These findings highlight the potential of MOF-derived carbon/graphene hybrids as a versatile and effective design strategy for high-performance energy-storage systems.

## Supplementary Material



## Notes

Cobalt nanoparticle size distribution in the MOF-derived porous carbon matrix (Figure S1); additional three-electrode CV curves of NPC, rGO, and NPC/rGO-20 at different scan rates (Figure S2); log­(i)-log­(v) linearization plots of anodic peak current versus scan rate for NPC, NPC/rGO-10, NPC/rGO-20, and rGO (Figure S3); additional GCD curves of NPC, rGO, and NPC/rGO-20 at different current densities (Figure S4); Ragone plot of the NPC/rGO-10//rGO asymmetric supercapacitor (Figure S5); comparative full-cell performance table against representative literature reports (Table S1); and the equations to calculate key parameters for three-electrode and device configurations (eqs S1–S6) (PDF)
