# Supplementary material for: Co-BDC MOF/Graphene Nanohybrid as an Efficient Electrode Material for High-Performance Supercapacitors
Source: ACS Omega. 2026 Mar 13;11(11):17732–45. doi: 10.1021/acsomega.5c11771 (PMC13019268; doi:10.1021/acsomega.5c11771)
Supplement: Supplementary file 1 [file ao5c11771_si_001.pdf]

Supporting information

Ana L. Braga[a], Tomaz A. S. Lima[a], Victor D. S. Fortunato[a], Danielle D. Justino[a,b], Pedro G. R. Gomes[e], Rayane C. F. Silva[b,c], Larissa F. M. A. Vieira[a], Hélio Ribeiro[d], Ana Paula C. Teixeira[b,c], Paulo F. R. Ortega[e], Rodrigo L. Lavall[b,c], Raquel V. Mambrini[a*], João Paulo C. Trigueiro[a,c*]

[a]Departamento de Química, Centro Federal de Educação Tecnológica de Minas Gerais, CEFET-MG, Belo Horizonte, MG, Brasil, 30480-000

[b]Departamento de Química, Instituto de Ciências Exatas, Universidade Federal de Minas Gerais, UFMG, Belo Horizonte, MG, Brasil, 31270-901

[c]Centro de Tecnologia em Nanomateriais e Grafeno CTNano/UFMG, Universidade Federal de Minas Gerais, UFMG, Belo Horizonte, MG, Brasil, 31310-270

[d]Departamento de Engenharia de Materiais, Escola de Engenharia, Universidade Presbiteriana Mackenzie, São Paulo, SP, Brasil

[e]Departamento de Química, Centro de Ciências Exatas, Universidade Federal de Viçosa, UFV, Viçosa, MG, Brasil, 36570-900

*Corresponding authors

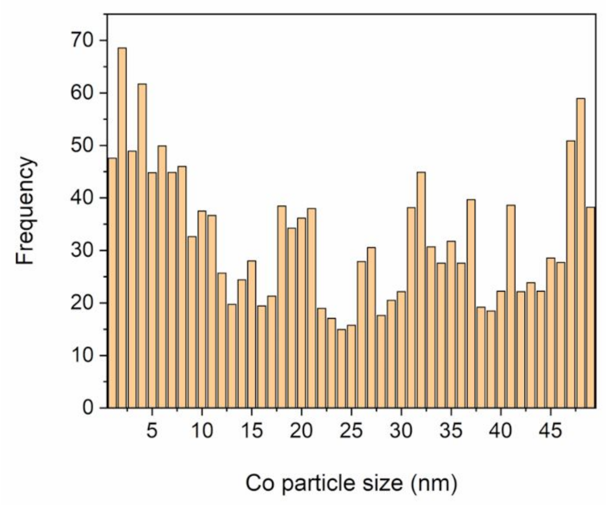

Figure S1. Nanoparticles size distribution of cobalt embedded in the MOF-derived porous carbon matrix.

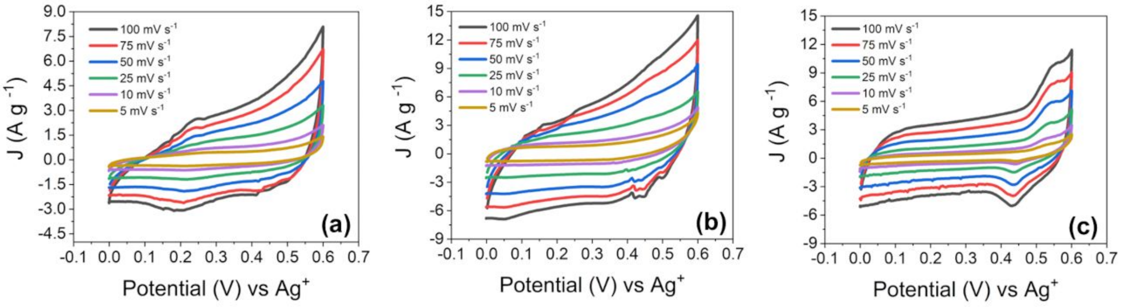

Figure S2. CV curves of NPC (a), rGO (b) and NPC/rGO-20 (c) in a 3-electrode cell in different scan rates.

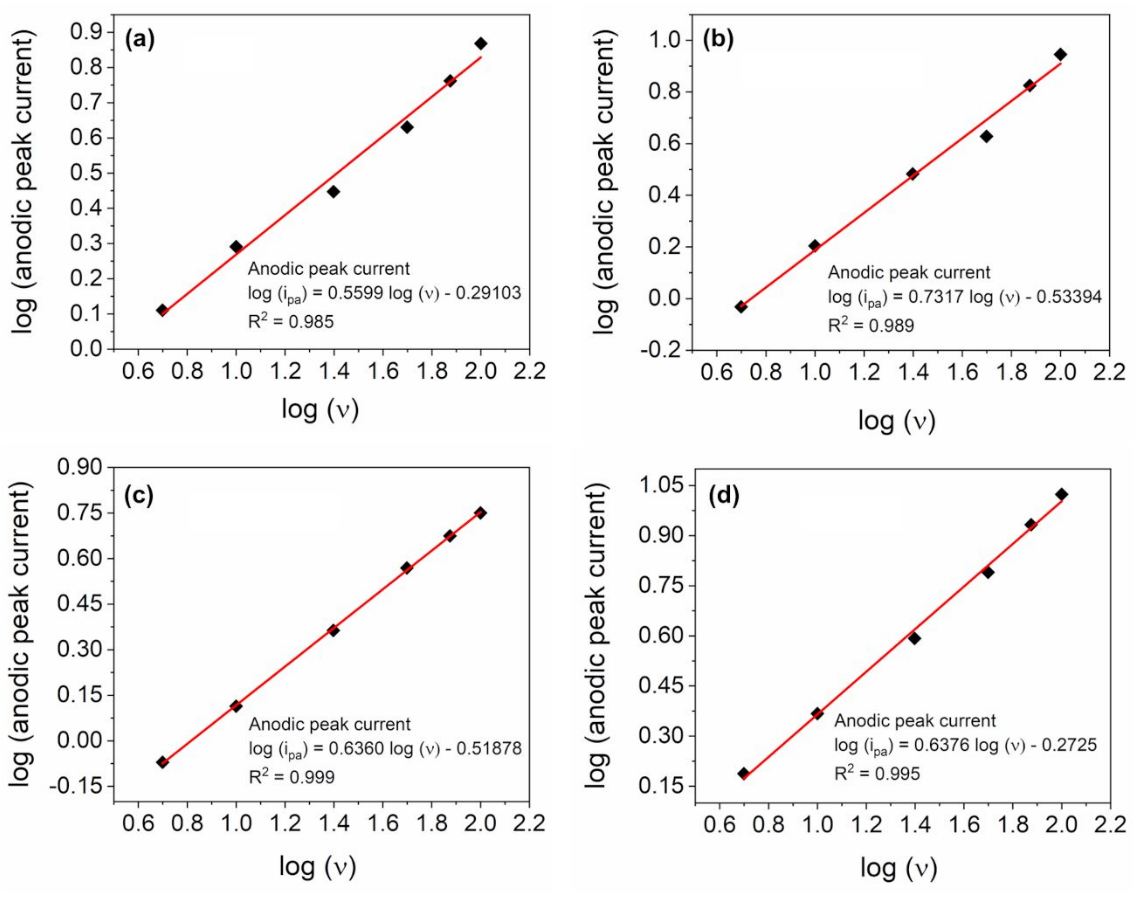

Figure S3. Fig. S5. Linearization of the logarithm of anodic peak currents versus the logarithm of scan rates for NPC (a), NPC/rGO-10 (b), NPC/rGO-20 (c) and rGO (d).

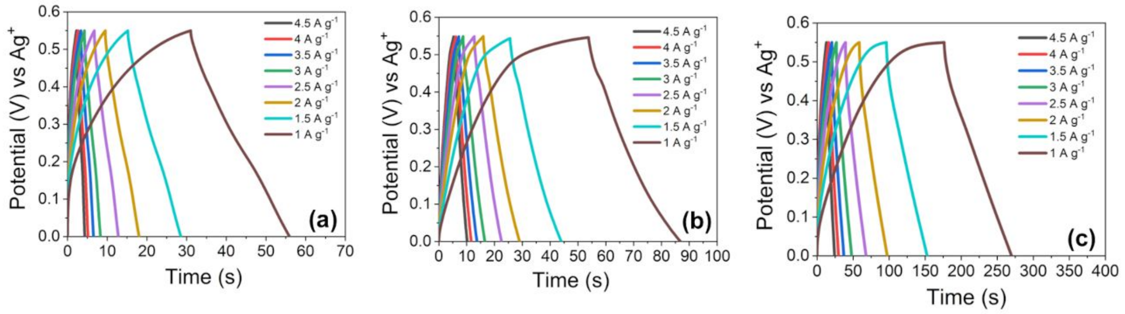

Figure S4. GCD curves of NPC (a), rGO (b) and NPC/rGO-20 (c) at different current densities.

The device demonstrates a energy density of 0.83 Wh kg⁻¹ at 1 A g⁻¹, corresponding to a power density of 241.0 W kg⁻¹ at 1 A g⁻¹ as shown in the Ragone plot.

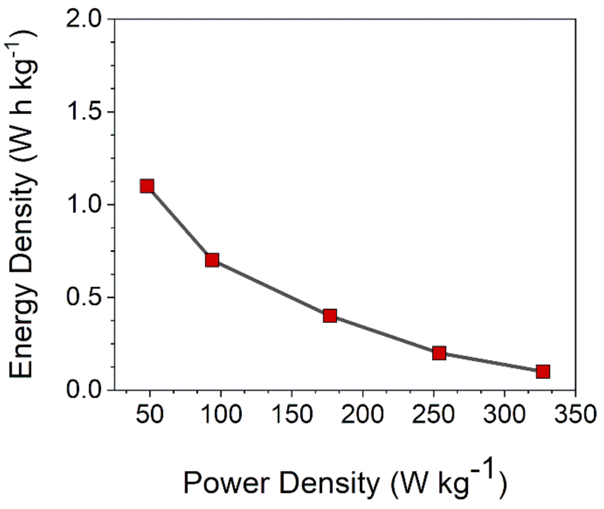

Figure S5. Ragone plot for asymmetric supercapacitor based on NPC/rGO-10//rGO.

Table S1. Electrochemical performance of full cells prepared for different composite materials

Material / Device (full cell)	***Specific capacitance (F g⁻¹)***	***Energy density (Wh kg⁻¹)***	***Power density (kW kg⁻¹)***	***Current density (A g⁻¹)***	***Potential window (V)***	***Cycle life / Retention***	***Reference***
NPC/rGO-10//rGO *(ASC) (this work)*	27.3	4.1	3.0	0.25	0–0.6	30,000 / 94.4 %	This work
Co@C // Co_3O_4@C(ASC)	17.9	5.6	0.38	1	0-1.5	1,000 / ~100 %	[1]
CoO / Co_3O_4 // AC (ASC)	38.6	10.52	0.14	0.2	0–1.4	30,000 / 133%	[2]
MIL-53(Al)@2%rGO // AC (ASC)	75	6.66	0.2	0.1	0–0.8	5,000 / 50 %	[3]
B-doped nanocarbons (SSC)	158	3.8	~0.17	1	0–1.0	----	[4]
GO-GNP-SiO_2 (SSC)	4.76	0.26	4	0.2	0–0.8	10 000 / ~92 %	[5]
$NiCo_2O_4$ // AC(ASC)	25	6.8	2.8	8	0–1.5	4,960 / ~85 %	[6]
MOF-derived porous carbon (SSC)	38.5	17.4	13.5	1	0–1.8	10,000 / 94.8 %	[7]

Pd-rGO/MOF // AC (ASC)	-	26.0	1.6	0.6–2.0	0.6	~3,500 / stable	[8]
$ZnCo_2O_4$ // MOF-derived NPC (ASC)	94.4	28.6	~0.10	0.1	~1.0	5,000 / 87.2 %	[9]
MIL-100(Fe)-derived carbon (ASC)	127.4	25.5	0.06	0.1	1.0	5,000 / 90.1 %	[10]

List of equations for the calculations of the supercapacitor and three electrode cells parameters.

$$Csp_{\,3\ electrodes} = \frac{2 \cdot I \cdot \int U dt}{(m_{we})(U^{i}_{discharge})^2}$$ (Eq. S1)

$$Csp = \frac{2 \cdot I \cdot \int V dt}{(m_+ + m_-)(V^{i}_{discharge})^2}$$ (Eq. S2)

$$ESR = \frac{V^{max}_{charge} - V^{max}_{discharge}}{I}$$ (Eq. S3)

$$\varepsilon = \frac{\Delta t_{discharge}}{\Delta t_{charge}} \times 100$$ (Eq. S4)

$$E = \frac{I \cdot \int V dt_{discharge}}{(m_+ + m_-)}$$

$$P = \frac{E}{(\Delta t_{discharge})}$$

(Eq. S6)

References

(1) Dai, E.; Xu, J.; Qiu, J.; Liu, S.; Chen, P.; Liu, Y. Co@Carbon and Co3O4@Carbon Nanocomposites Derived from a Single MOF for Supercapacitors. *Scientific Reports* **2017**, *7* (1), 12588. https://doi.org/10.1038/s41598-017-12733-5.

(2) Pang, M.; Long, G.; Jiang, S.; Ji, Y.; Han, W.; Wang, B.; Liu, X.; Xi, Y.; Wang, D.; Xu, F. Ethanol-Assisted Solvothermal Synthesis of Porous Nanostructured Cobalt Oxides (CoO/Co3O4) for High-

Performance Supercapacitors. *Chemical Engineering Journal* **2015**, *280*, 377–384. https://doi.org/10.1016/j.cej.2015.06.053.

(3) Majumder, M.; Choudhary, R. B.; Thakur, A. K.; Khodayari, A.; Amiri, M.; Boukherroub, R.; Szunerits, S. Aluminum Based Metal-Organic Framework Integrated with Reduced Graphene Oxide for Improved Supercapacitive Performance. *Electrochimica Acta* **2020**, *353*, 136609. https://doi.org/10.1016/j.electacta.2020.136609.

(4) Guo, H.; Gao, Q. Boron and Nitrogen Co-Doped Porous Carbon and Its Enhanced Properties as Supercapacitor. *Journal of Power Sources* **2009**, *186* (2), 551–556. https://doi.org/10.1016/j.jpowsour.2008.10.024.

(5) Zhu, C.; Liu, T.; Qian, F.; Han, T. Y.-J.; Duoss, E. B.; Kuntz, J. D.; Spadaccini, C. M.; Worsley, M. A.; Li, Y. Supercapacitors Based on Three-Dimensional Hierarchical Graphene Aerogels with Periodic Macropores. *Nano Letters* **2016**, *16* (6), 3448–3456. https://doi.org/10.1021/acs.nanolett.5b04965.

(6) Ding, R.; Qi, L.; Jia, M.; Wang, H. Facile and Large-Scale Chemical Synthesis of Highly Porous Secondary Submicron/Micron-Sized NiCo2O4 Materials for High-Performance Aqueous Hybrid AC-NiCo2O4 Electrochemical Capacitors. *Electrochimica Acta* **2013**, *107*, 494–502. https://doi.org/10.1016/j.electacta.2013.05.114.

(7) Yu, F.; Wang, T.; Wen, Z.; Wang, H. High Performance All-Solid-State Symmetric Supercapacitor Based on Porous Carbon Made from a Metal-Organic Framework Compound. *Journal of Power Sources* **2017**, *364*, 9–15. https://doi.org/10.1016/j.jpowsour.2017.08.013.

(8) Teffu, D. M.; Ramoroka, M. E.; Makhafola, M. D.; Makgopa, K.; Maponya, T. C.; Seerane, O. A.; Hato, M. J.; Iwuoha, E. I.; Modibane, K. D. High-Performance Supercabattery Based on Reduced Graphene Oxide/Metal Organic Framework Nanocomposite Decorated with Palladium Nanoparticles. *Electrochimica Acta* **2022**, *412*, 140136. https://doi.org/10.1016/j.electacta.2022.140136.

(9) He, D.; Gao, Y.; Yao, Y.; Wu, L.; Zhang, J.; Huang, Z.-H.; Wang, M.-X. Asymmetric Supercapacitors Based on Hierarchically Nanoporous Carbon and ZnCo2O4 From a Single Biometallic Metal-Organic Frameworks (Zn/Co-MOF). *Frontiers in Chemistry* **2020**, *Volume 8-2020*. https://doi.org/10.3389/fchem.2020.00719.

(10) Kim, S. C.; Choi, S. Q.; Park, J. Asymmetric Supercapacitors Using Porous Carbons and Iron Oxide Electrodes Derived from a Single Fe Metal-Organic Framework (MIL-100 (Fe)). *Nanomaterials* **2023**, *13* (12). https://doi.org/10.3390/nano13121824.
